# A unified constitutive model for pressure sensitive shear flow transitions in moderate dense granular materials

**DOI:** 10.1038/s41598-021-99006-4

**Published:** 2021-10-04

**Authors:** Xiaohui Cheng, Shize Xiao, Alex Sixie Cao, Meiying Hou

**Affiliations:** 1 Department of Civil Engineering, Tsinghua University, Beijing, China; 2 Department of Structural Engineering, Norwegian University of Science and Technology, Trondheim, Norway; 3 Institute of Physics, Chinese Academy of Science, Beijing, China

**Keywords:** Engineering, Materials science, Physics

## Abstract

Granular shear flows exhibit complex transitional regimes that are dramatically affected by the pressure level and shear stress state. New advances in granular shear tests at low pressure have enlightened the understanding of the two granular shear flow transitions: between quasi-static and moderate shear flows, and between steady-state and transient shear flows. However, a unified constitutive model to describe these two transitions is yet to develop. In this work, a simplified and unified model is proposed based on innovative triaxial shear flow tests, using two dimensionless physical variables. Model results validated against experimental data suggest that the shear flow transition between a quasi-static to a moderate Isotach type flow state is highly pressure-dependent. At extremely low pressure, the granular viscosity becomes the primary mechanism, suppressing the quasi-static mechanism even under “quasi-static” shear rates. In transient to steady state granular flow transitions, a mobilized shear stress ratio or mobilized friction coefficient between zero and the critical state ratio for consolidated granular packings is taken into consideration. This is coupled with the mechanism of granular viscosity. These findings have not been discussed before and are of great relevance to granular mechanics as well as space and earthquake engineering.

## Introduction

Constitutive models for granular shear flows with practical engineering relevance have received increasing attention in scientific and engineering fields in recent years^1–4^. Landslides and subsidence caused by earthquakes or other dynamic impacts have caused tremendous damage. Such granular flow transitions threaten vital marine infrastructure such as submarine pipelines and cables, foundations for various offshore structures, and submerged tunnels. Current engineering practices rely heavily on engineering experience, which is in many cases insufficient. There is also no accepted method to estimate the dynamic forces of granular flows on buried structures during and post-earthquake^5^. Seismic resilient design considering large post-liquefaction deformations has received much attention. It has required increasingly more fundamental research on granular flow transitions or phase transitions under low pressure conditions to accurately describe and predict post-liquefaction behavior.

In space exploration, the rate-dependent behavior of granular materials in microgravity or low confining pressure conditions is important. With accurate constitutive models in microgravity, impact forces from landing gear can be predicted accurately and the design of such landing gear can be optimized. Liquefaction caused by the pressure increase of trapped gas in lunar regolith has also been observed on the Moon^6,7^. These observations have made research on liquefaction properties of lunar regolith or simulants in microgravity important. In particular, the large peak friction angle of sand under microgravity conditions observed in NASA-MGM experiments^8^ need further understanding from the perspective of granular physics in low gravity or pressure conditions.

Scientists within the field of geotechnical engineering have proposed some engineering models for the quasi-static response of sand in pre-critical states. The critical state is a material state in which a steady plastic shear flow is assumed. Among these models, elastoplastic theory^9^ and empirical models^10^ based on extensive experimental data has been found to be representative. However, constitutive models that represent transitional behavior between quasi-static and moderate shear flows, and between steady-state and transient shear flows are yet to develop.

For Isotach clays subjected to an instantaneous increase in strain rates, the stress path will increase instantaneously and follow a new and higher stress path. Such an Isotach behavior of clay and its underlying mechanism of pore water viscosity are mostly absent in sand and other granular materials^11^. Recent findings suggest that more complicated viscosity behavior of granular materials such as TESRA and P&N behavior, are present^12^. It is not uniformly agreed upon which fundamental mechanisms such as viscosity, friction, or others, are correct. The occurrence of transitions between TESRA and Isotach behavior are also of question.

A unified constitutive model that can describe the granular flow transitional behavior from a quasi-static state to a moderate flow state is urgently needed. The constitutive models representing quasi-static stress–strain relationships in sands are mainly divided into elastoplastic and nonlinear “elastic” models. An elastoplastic model is the critical Camclay model^13^, which uses a specific yield surface, plastic flow criterion, and hardening criterion. Nonlinear “elastic” models are often empirical because of their simple form, which include Duncan-Zhang^14^, K-G^15^, and others. Theoretical Cauchy and Green nonlinear elastic incremental models are also available, though used less frequently^16^.

In general, nonlinear empirical models represent the quasi-static behavior in sand or other granular materials through the relationship between mobilized shear stress ratio or mobilized friction coefficient and strain. For very small shear strains of less than 10^−5^, granular materials are generally in a pure elastic state and the nonlinear shear modulus is constant. In the elastic state, the small-strain shear modulus is affected by volume fraction, effective stress, particle size distribution, and particle shape. When the shear strain increases, the nonlinear shear modulus degrades continuously until it becomes almost zero for very large shear strains. In this state, the granular material has reached the critical state where it can be sheared continuously with a constant shear stress. Oztoprak and Bolton^17^ proposed an empirical model for the nonlinear shear modulus by evaluating extensive experimental data of sand stiffness.

The research group MiDi (Groupement de Recherche Milieux Divisés)^1,2^ established the $\mu \left( I \right)$ constitutive rheological model for steady dense granular flows based on extensive experiments and numerical simulations. This model can describe steady flow behavior of granular materials for moderate strain-rates by using the inertial number $I$, a dimensionless relative strain rate, as a variable. This model was used as the plastic flow rule in order to formulate a nonlinear elastic and perfectly viscoplastic model for dense granular flows^3^. However, it fails to represent transient to steady flow transitions of granular materials.

In space and earthquake engineering problems, sands or other granular materials are often in a quasi-static mobilized stress state far below the critical quasi-static stress state when subject to static loads with a load around one third to one fourth of the critical load. When granular materials are subject to seismic or dynamic loads such as earthquakes and landslides, transient flows are more likely to occur than steady flows. Such transient flows include creep or decreased flow and creep rupture or accelerated flow. Neither the MiDi model nor quasi-static soil mechanics models are suitable in this case.

A series of laboratory tests on sands at low effective isotropic stress were systematically performed to reveal transient flows^8,18–20^. Results from laboratory tests reveal a significant S-shape between strain rates and viscous shear stresses. This behavior is not captured by the rate-dependent Bingham model nor by rate-independent critical state soil mechanics models. Moreover, the underlying mechanism is not yet known, and the pressure sensitive characteristics of such transient flows is yet to be investigated^21,22^.

In this paper, a novel constitutive model is proposed based on advanced triaxial shear flow tests under the conditions of isotropy and moderate density of the sample, using two dimensionless physical variables: the mobilized friction coefficient and the inertial number. The mobilized friction coefficient is used to capture the transient to steady flow transition of granular materials starting from any quasi-static noncritical state. Its development can be formulated in terms of the shear strain variation. The inertial number is adopted to characterize the rate and pressure dependency of granular flows. The model is validated against experimental data and the results suggest that the shear flow transition between a quasi-static to a moderate Isotach type flow state is highly pressure-dependent. At extremely low pressure, the granular viscosity becomes the primary mechanism, suppressing the quasi-static mechanism even under “quasi-static” shear rates. In transient to steady state granular flow transition, the mobilized shear stress ratio between zero and the critical state ratio for any consolidated granular packings should be taken into consideration which interacts with the mechanism of granular viscosity. Predictions and analysis based on the proposed model are done to provide an understanding of the effects of low effective isotropic stresses and shear rates on granular materials with an initial moderate density. Pressure sensitive and rate-dependent transient flow characteristics of granular materials are disclosed for the first time.

## Model development

### Conceptual framework

**Figure 1:**
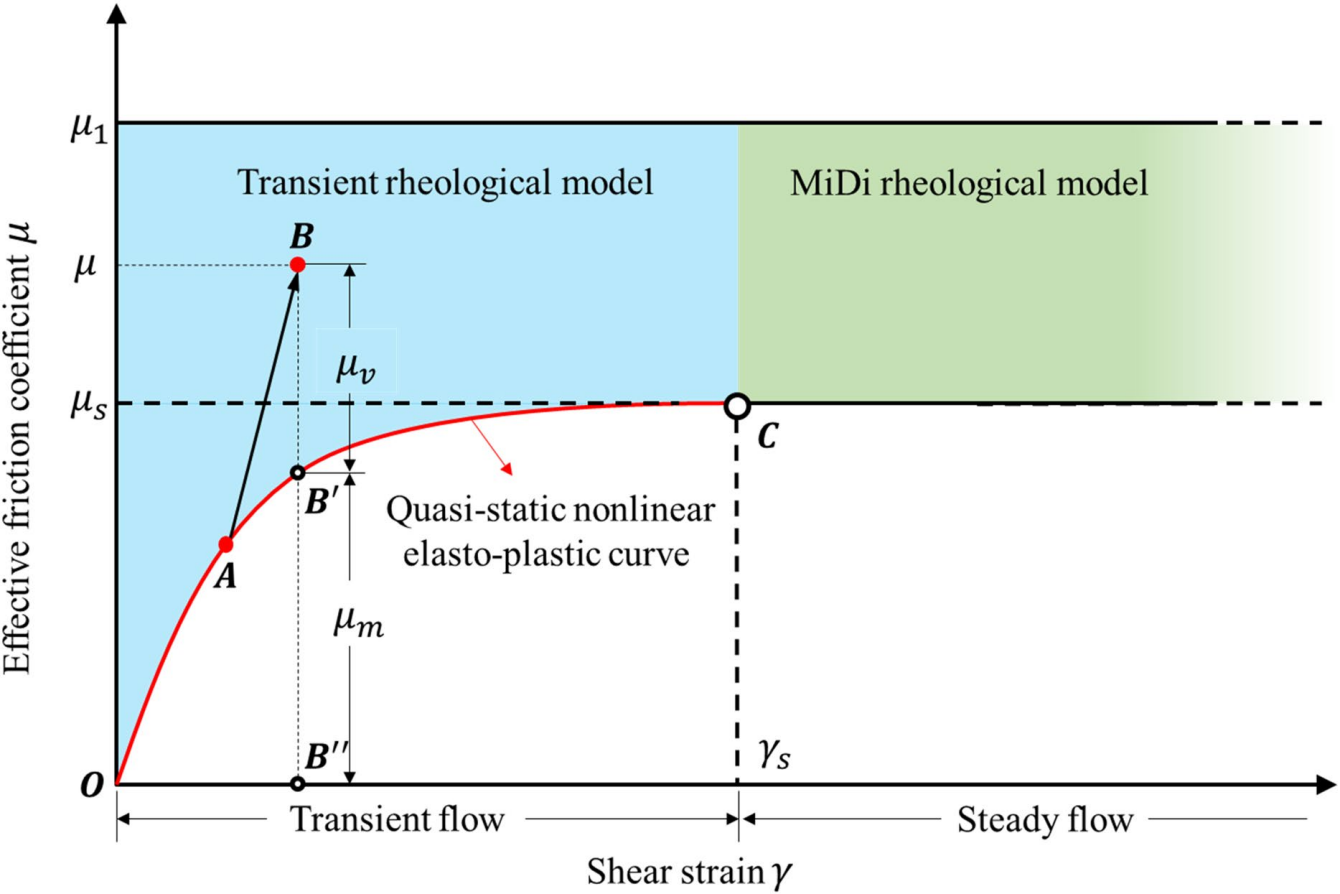
Components of the effective friction coefficient $\mu = \tau /p^{\prime }$ in various states of flow of granular materials. The red line $\widehat{OC}$ is the quasi-static stress–strain relation described by soil mechanics in the solid-like quasi-static domain. The green area is the domain of steady state flow described by the MiDi rheological model. The blue area is the domain of transient flow described by the transient rheological models.

Figure 1 shows the relationship between the shear strain $\gamma$ and the effective friction coefficient $\mu$ and its components. This shear stress–strain plane with shear flow rate effects is commonly used to characterize the shear flow behavior of sand and other granular materials. The upper flat line $\mu_{1}$ signifies the threshold in the granular material between a steady dense moderate flow for $\mu < \mu_{1}$ and an accelerated unsteady dense flow for $\mu > \mu_{1}$. In the latter case, which is beyond the scope of this paper, the long-term contact between particles is no longer maintained and binary collision becomes the main mechanism of momentum exchange. The lower curved line $\widehat{{{\text{OC}}}}$ is the quasi-static mobilized friction coefficient $\mu_{m}$. The mobilized friction coefficient $\mu_{m}$ represents the stress–strain relation in a quasi-static state and its upper bound $\mu_{s}$ is the critical state of Drucker-Prager yield surface. It signifies the threshold in the granular material between a quasi-static state for $\mu < \mu_{m}$ and a moderate shear flow for $\mu > \mu_{m}$.

In a steady state when $\mu > \mu_{s}$ and $\gamma > \gamma_{s}$, the viscous stress in a steady dense moderate flow is believed to be a function of flow rate $\dot{\gamma }$ and effective isotropic stress $p^{\prime }$. For most of the cases in seismic and dynamic engineering, the soils are statically loaded in the transient domain at point A and not in the steady domain at point C in Fig. 1. Thus, the granular materials cannot be characterized by a steady shear flow and will mainly inherit properties of a transient shear flow.

The concept for granular materials is analogous with the concept of phase transitions for matters such as water, but the scale in which the particles interact are different. In water, viscosity is determined by intermolecular forces such as van der Waals forces and hydrogen bonds. In granular matters, it is thought that the main intergranular mechanism is friction. The inertial number $I$ is used to characterize the granular friction-based viscosity, which is related to the change of intrinsic time scale or frequency of the granular flow due to different effective isotropic stress levels.

In this paper, three friction coefficients have been introduced, namely the critical friction coefficient $\mu_{s}$, the upper limit friction coefficient $\mu_{1}$ and the mobilized friction coefficient $\mu_{m}$. Of the three mentioned friction coefficients, the upper limit friction coefficient $\mu_{1}$ is not well known in granular mechanics. In fluid mechanics and granular physics, the mobilized friction coefficient $\mu_{m}$ is not well known. The mobilized friction coefficient $\mu_{m}$ is the quasi-static friction coefficient of a granular material in any pre-consolidated state, or for any effective isotropic stress $p^{\prime }$. When a granular material with zero deviatoric stress $\tau$ is quasi-statically sheared from a pre-consolidated isotropic state to a critical state, the mobilized friction coefficient $\mu_{m} = \mu_{m} \left( {p^{\prime } ,\gamma } \right)$ gradually develops from zero to the critical friction coefficient $\mu_{s}$.

### Mathematical formulation

The original MiDi model in tensor form assumes granular flows incompressible and is expressed as Eqs. (1) and (2):

1 $$ \sigma_{ij} = p^{\prime } \delta_{ij} + \tau_{ij} $$

2 $$ \tau_{ij} = \mu \left( I \right)p^{\prime } \frac{{\dot{\gamma }_{ij} }}{{\dot{\gamma }}} $$

with $\mu \left( I \right)$ and $I$ defined in Eqs. (3) and (4):

3 $$ \mu \left( I \right) = \mu_{s} + \frac{{\mu_{1} - \mu_{s} }}{{\frac{{I_{0} }}{I} + 1}} $$

4 $$ I = \dot{\gamma }d\sqrt {\frac{{\rho_{s} }}{{p^{\prime } }}} = \frac{{T_{p} }}{{T_{\gamma } }} $$

In the above equations, $\mu \left( I \right)$ is the rate-dependent and pressure dependent friction coefficient, $p^{\prime}$ is the effective isotropic stress or the effective hydrostatic stress, $\dot{\gamma }_{ij}$ is the deviatoric strain rate tensor and $\dot{\gamma } = \sqrt {\dot{\gamma }_{ij} \dot{\gamma }_{ij} /2}$ is the second invariant of $\dot{\gamma }_{ij}$. In Eqs. (3) and (4), $\mu_{s} = \tan \theta_{s}$ is the critical friction coefficient in a quasi-static state, $\mu_{1} = \tan \theta_{1}$ is the upper limit friction coefficient, $I_{0}$ is a material parameter reflecting the rheological properties of the granular material, $I$ is the inertial number and $d$ is the mean particle diameter. The inertial number can be expressed as the ratio between the microscopic time scale $T_{p} = d/\sqrt {p^{\prime } /\rho_{s} }$ and the macroscopic time scale $T_{\gamma } = \dot{\gamma }^{ - 1}$. The microscopic time scale $T_{p}$ represents the time required for a particle to undergo a displacement of the mean particle diameter under an effective isotropic stress $p^{\prime }$. The macroscopic time scale $T_{\gamma }$ represents the time required to generate a unit of strain.

In this paper, an extension and modification of the MiDi model is formulated by separating the deviatoric stress $\tau_{ij}$ into a rate-independent quasi-static component $\tau_{ij}^{s}$ and a rate-dependent viscous component $\tau_{ij}^{v}$ as shown in Eq. (5):

5 $$ \tau_{ij} = \tau_{ij}^{s} + \tau_{ij}^{v} $$

In the quasi-static component $\tau_{ij}^{s}$, the critical friction coefficient $\mu_{s}$ is replaced by the mobilized friction coefficient $\mu_{m}$ with an upper bound limit of $\mu_{m} \le \mu_{s}$ as shown in Eq. (6):

6 $$ \mu_{m} = \frac{{\tau^{s} }}{{p^{\prime } }} \le \mu_{s} $$

where $\tau^{s} = \sqrt {\tau_{ij}^{s} \tau_{ij}^{s} /2}$ is the second invariant of $\tau_{ij}^{s}$. In the original MiDi model, the direction of the critical shear stress is associated with the direction of the deviatoric strain rate $\dot{\gamma }_{ij}$. This is corrected in the present model as the static shear stress $\tau_{ij}^{s}$ is a function of the nonlinear shear modulus $G$ and deviatoric strain tensor $\gamma_{ij}$ as shown in Eq. (7):

7 $$ \tau_{ij}^{s} = G\gamma_{ij} $$

where $G$ is the shear modulus. $\tau_{ij}^{v}$ is extended to include transient flows as shown in Eq. (8).

8 $$ \tau_{ij}^{v} = \frac{{\mu_{1} - \mu_{m} }}{{\frac{{I_{0} }}{I} + 1}}p^{\prime } \frac{{\dot{\gamma }_{ij} }}{{\dot{\gamma }}} $$

We propose to calculate the quasi-static deviatoric stress by using the nonlinear expression for the shear modulus $G$ proposed by Oztoprak and Bolton^17^. The nonlinear shear modulus can be expressed as Eq. (9):

9 $$ G = \frac{{A\left( \gamma \right)p_{a} }}{{\left( {1 + e} \right)^{3} }}\left( {\frac{{p^{\prime } }}{{p_{a} }}} \right)^{m\left( \gamma \right)} $$

where $p_{a}$ is the standard atmospheric pressure, $e$ is the void ratio, $p^{\prime }$ is the effective isotropic stress and $A\left( \gamma \right)$ and $m\left( \gamma \right)$ are fitting parameters that are dependent on the shear strain $\gamma$.

We also propose that $A\left( \gamma \right)$ and $m\left( \gamma \right)$ can be expressed as Eqs. (10) and (11):

10 $$ A\left( \gamma \right) = \frac{{A_{0} }}{{1 + \frac{\gamma }{{\gamma_{r2} }}}} $$

11 $$ m\left( \gamma \right) = m_{0} + \frac{{m_{1} - m_{0} }}{{1 + \frac{{\gamma_{r1} }}{\gamma }}} $$

12 $$ \gamma_{r2} = \frac{{M\left( {1 + e} \right)^{3} }}{{\sqrt 3 A_{0} }} $$

where $m_{0}$, $m_{1}$ and $A_{0}$ are parameters directly determined from experimental results, $\gamma_{r1}$ and $\gamma_{r2}$ are reference strain levels and $M = \mu_{s} /\sqrt 3$ is the critical stress ratio in a triaxial compression test. $\gamma_{r1}$ can be obtained from the curve fitting of $m\left( \gamma \right)$, and $\gamma_{r2}$ can be determined from Eq. (12). From the extreme cases of $G\left. \right|_{\gamma \to 0} = G_{0}$ and $G\left. \right|_{{\gamma \to \gamma_{s} }} = 0$, Eq. (9) can be calibrated to successfully predict the nonlinear behavior of the shear modulus $G$ of the sand sample with an initial moderate density and subjected to unrotated principal stress conditions. Since the experimental database established by Oztoprak and Bolton does not include data for effective isotropic stresses of $p^{\prime } < 10\;{\text{kPa}}$, the applicability of the model for such effective isotropic stresses must be verified. In this paper, it is assumed that the model is still valid for low effective isotropic stresses according to^23^.

**Figure 2:**
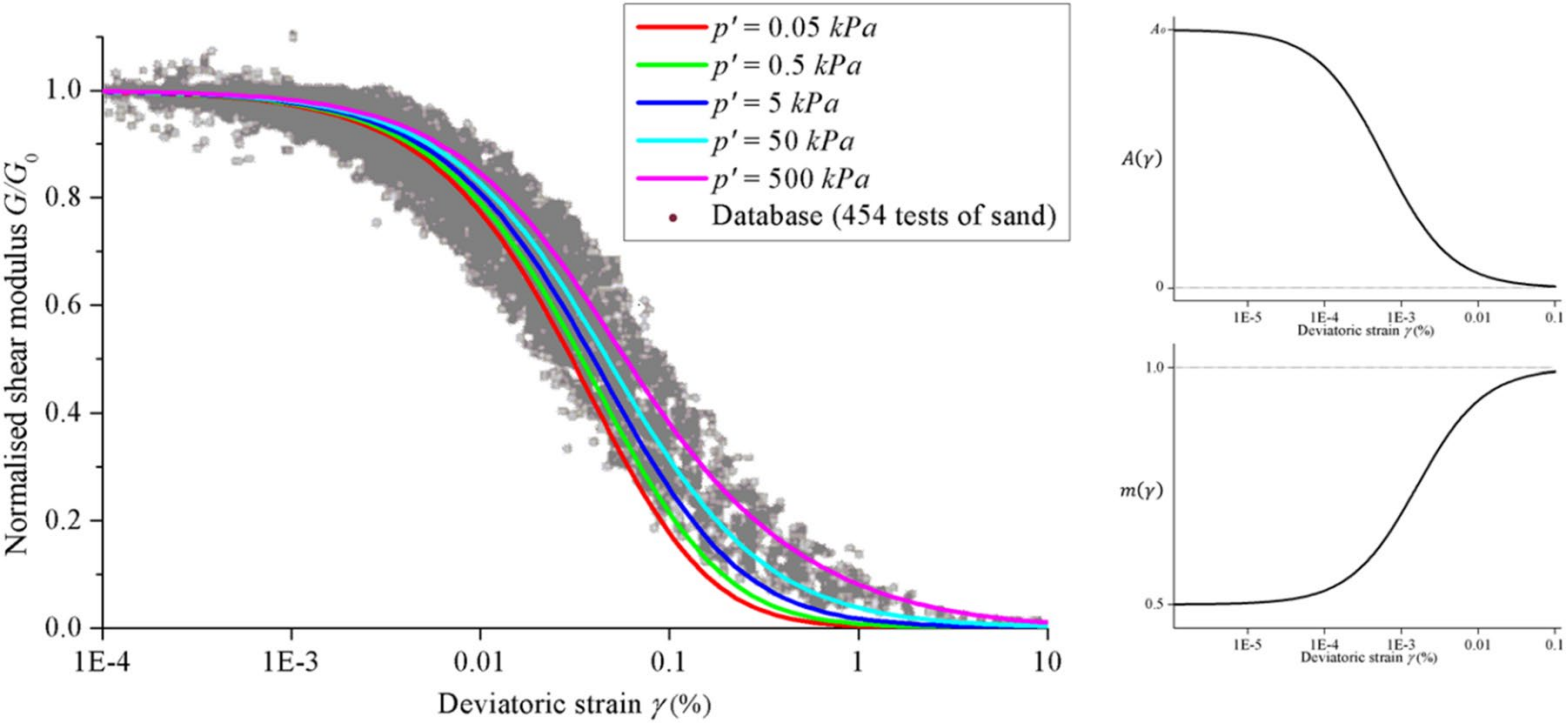
Oztoprak and Bolton’s nonlinear shear modulus plotted together with extensive test data, and model fitting parameters $m\left( \gamma \right)$ and $A\left( \gamma \right)$^17^.

In Fig. 2, observe that Oztoprak and Bolton’s expression for the nonlinear shear modulus correlates well with experimental data. Thus, it can be deemed to represent the behavior accurately.

### Model characteristics

The proposed model described in “Mathematical formulation” section, is a simple unified constitutive model for incompressible granular flows starting from any quasi-static stress state, including the critical state. This model implies that the total deviatoric stress consists of a quasi-static nonlinear elastic component $\tau_{ij}^{s}$ and a viscous component $\tau_{ij}^{v}$. In a triaxial setup, when $i \ne j$, $\sigma_{ij} = 0$, $\gamma_{ij} = 0$, $\sigma_{22} = \sigma_{33}$, and $\gamma_{22} = \gamma_{33}$. The second invariants of $\tau_{ij}$ and $\gamma_{ij}$ can be simplified to $\tau = \left( {\sigma_{11} - \sigma_{33} } \right)/\sqrt 3$, and $\gamma = \left( {\gamma_{11} - \gamma_{33} } \right)/\sqrt 3$ respectively. The deviatoric stress can be expressed as Eq. (13):

13 $$ q = \sigma_{11} - \sigma_{33} = \sqrt 3 \tau . $$

The quasi-static component and viscous component can be found by reducing Eqs. (6)–(8) to (14) and (15):

14 $$ q^{s} = G\left( {\gamma_{11} - \gamma_{33} } \right) = \sqrt 3 G\gamma {,} $$

15 $$ q^{v} = \frac{{\mu_{1} - \mu_{m} }}{{\frac{{I_{0} }}{I} + 1}}p^{\prime } \frac{{\left( {\dot{\gamma }_{11} - \dot{\gamma }_{33} } \right)}}{{\dot{\gamma }}} = \sqrt 3 \frac{{\mu_{1} - \mu_{m} }}{{\frac{{I_{0} }}{I} + 1}}p^{\prime } , $$

16 $$ \mu = \frac{\tau }{p^{\prime}} = \mu_{m} + \frac{{\mu_{1} - \mu_{m} }}{{\frac{{I_{0} }}{I} + 1}} = \mu_{m} + \mu_{v} , $$

where $\mu_{v}$ is the viscous friction coefficient. Together, the quasi-static component $q^{s}$ and the viscous component $q^{v}$ can describe transient granular flows under the conditions of isotropy and moderate density of the sample starting from any quasi-static stress state but the critical state.

## Simulations of transient granular flows

### Multi-stage triaxial creep tests in low effective isotropic stresses

To investigate post-liquefaction flow mechanisms of sand, Towhata et al.^19^ performed triaxial experiments on sand for low effective isotropic stresses $p^{\prime }$. A specific multi-stage creep stress path was used to obtain the correlation between the transient strain rate $\dot{\gamma }$ and the driving viscous stress $q^{v}$. In every loading stage, the discrepancy between the loading state and the quasi-static or reference curve is the viscos stress component $q^{v}$. As creep develops at each load stage, the quasi-static component $q^{s}$ increases with the development of shear strain $\gamma$, and the viscous component $q^{v}$ decreases as the shear strain rate $\dot{\gamma }$ decreases.

Although the triaxial setting has the advantage of investigating granular transient flow studies under controlled effective isotropic pressure, it cannot introduce the stress and strain-rate tensor rotation. However, a triaxial setting allows us to achieve an insight into the pressure sensitive granular flows. In this paper, numerical simulations of the two kinds of triaxial experiments are performed. General material parameters from Towhata et al.^19,20^ are used and model specific parameters are derived from experimental data. The critical stress ratio $M$ is obtained in the critical state from the data set obtained by Been et al.^24^, $A_{0}$ is determined by measuring the small-strain shear modulus $G_{0}$ in Eq. (9) for $\gamma \to 0$, and $\gamma_{r1}$ and $\gamma_{r2}$ are calibrated to fit the quasi-static stress–strain relationship. The parameters used in the numerical simulations are shown in Table 1, where $M$ and $\mu_{1}$ are material constants, and $I_{0}$ is a state related parameter.

**Table 1:** Material parameters used for simulations in this paper.

	Yurakucho sand	Miho sand	Toyoura sand
**General**			
Minimum void ratio *e*_*min*_	0.780	0.571	0.678
Maximum void ratio* e*_*max*_	1.268	0.938	0.992
Relative density *D*_*r*_	30.6%	85.1%	87.5%
Average particle diameter *d* (mm)	0.240	0.161	0.196
Particle density $\rho_{s}$ (kg/m^3^)	2700	2663	2640
**Specific**			
Critical stress ratio $M$	2.01	2.267	1.82
Fitting parameter $A_{0}$	2400	1105	1600
Fitting parameter $\gamma_{r1}$	0.15%	0.20%	0.10%
Fitting parameter $\gamma_{r2}$	0.48%	0.51%	0.33%
Fitting parameter $I_{0}$	4 × 10^−7^	4 × 10^−7^	4 × 10^−9^
Fitting parameter $\mu_{1}$	1.428	1.235	1.068

**Figure 3:**
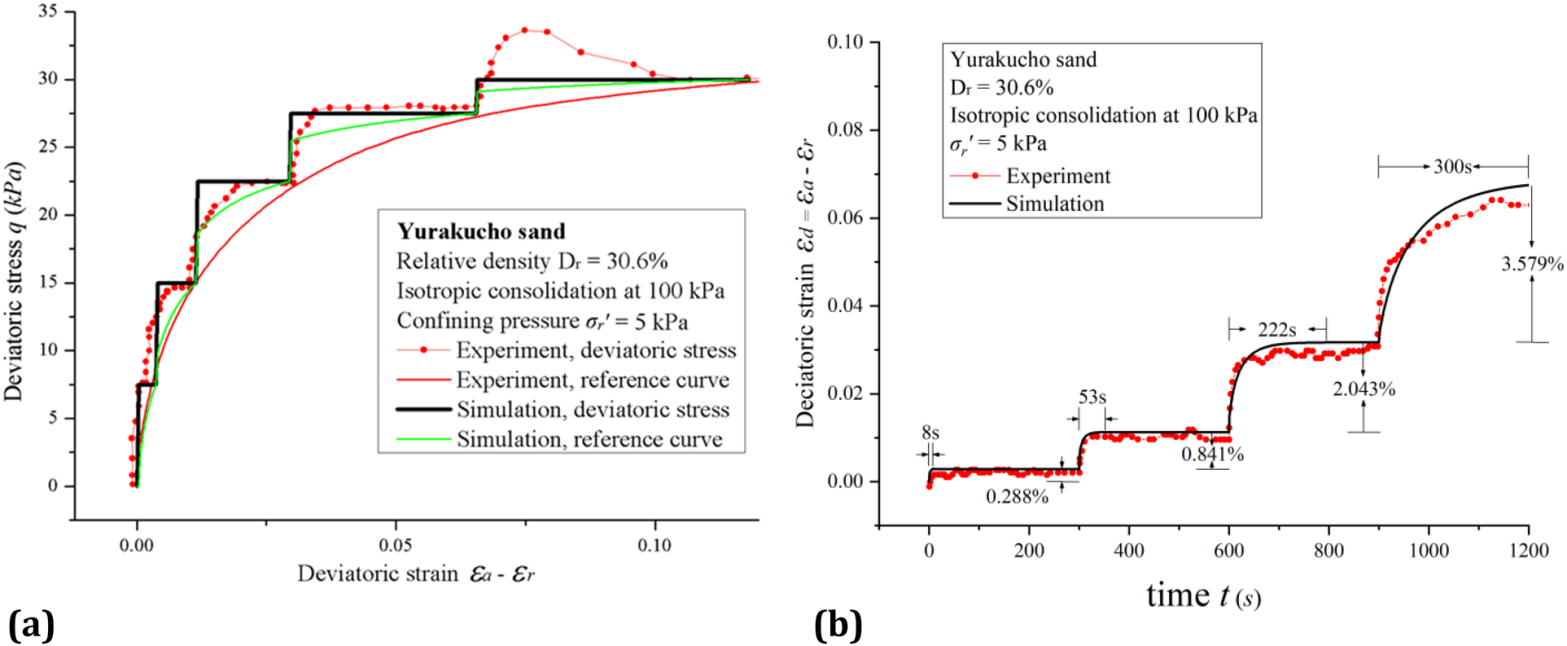
Experimental and simulation results of triaxial compression experiments on Yurakucho sand ((**a**) Simulation of deviatoric stress–strain curve; (**b**) Simulation of time-deviatoric strain curve with loading interval of 300 s).

Figure 3 show experimental and simulation results of monotonic multi-stage triaxial creep flow on Yurakucho sand. In Fig. 3a, the red dotted line is the measured deviatoric stress, the red solid line is the quasi-static reference curve of experiment data, the black solid line is the simulated deviatoric stress, and the green solid line is the simulated reference curve. In Fig. 3b, the red dotted line is the experimental line and the black solid line is the simulated time-deviatoric strain curve. In the experiment, the reference curve represents the quasi-static stress–strain relationship defined by the stress–strain points at the end of each loading step.

In Fig. 3a, large deviatoric step loads ${\Delta }q$ are applied. We assume that the experiment sample reaches a quasi-static state when the deviatoric strain rate $\dot{\gamma }$ is less than $10^{ - 8} \;{\text{s}}^{ - 1}$. For constant deviatoric strains $\gamma$, the sudden application of each deviatoric step load ${\Delta }q$ will result in a sudden increase of $q$ due to the sudden increase of the effective isotropic stress $p^{\prime }$.These effects cause the jump and following derivation in the simulated reference curve according to Eq. (14) compared to experimental reference curve that is simply smooth connection of ending points of each load step. The experiments did not accurately maintain a constant stress which is the source of the discrepancy between the experimental and simulated deviatoric stress curve.

In Fig. 3b, the deviatoric strain $\gamma$ develops rapidly after the application of every step load $\Delta q$. The deviatoric strain $\gamma$ appears to reach a steady state at each loading step after sufficient time has passed. The development of the deviatoric strain $\gamma$ may be explained through the interplay between the quasi-static component $q^{s}$ and the viscous component $q^{v}$. When a deviatoric stress step ${\Delta }q$ is applied to any quasi-static state $q^{s}$. A transient viscous component $q^{v} = {\Delta }q - {\Delta }q^{s}$ is generated. Due to the nonzero viscous component $q^{v} \ne 0$, deviatoric strain $\gamma$ develops. Because of the increase in deviatoric strain $\gamma$, the quasi-static component $q^{s}$ in the sample increases gradually towards its critical value $q_{c}^{s}$ and the viscous component $q^{v}$ decreases in the same manner. This leads to the increase of the deviatoric strain $\gamma$ with a decreasing deviatoric strain rate $\dot{\gamma }$. Eventually, the sample reaches a quasi-static state.

The creep time and strain in each loading step increases stepwise. This can largely be explained by the shear modulus $G$. When the deviatoric stress $q$ is small, the shear modulus $G$ is large and the strain increment ${\Delta }\gamma$ is small. When the deviatoric stress $\tau$ is higher, the deviatoric strain increment ${\Delta }\gamma$ becomes larger for similar deviatoric stress increments ${\Delta }q$. Increasing deviatoric strain ${\Delta }\gamma$ for increasing values of the deviatoric stress $q$ means that more creep time is needed for the sample to reach a new quasi-static state.

The simulation and experimental results shown in this section reflect the correlation between the viscous component $q^{v}$ and the deviatoric strain rate $\dot{\gamma }$ in the sample, and that rate-dependency is affected by the effective isotropic stress $p^{\prime}$. In the following sections, We will present predictions and analyze the rate-dependent properties of sandy materials at different effective isotropic stress states $p^{\prime}$ and deviatoric strain rates $\dot{\gamma }$. These properties are represented through the ratio $q^{v} /q_{c}^{s}$ and the deviatoric strain rate $\dot{\gamma }$.

The main factors influencing the viscous component $q^{v}$ are the effective isotropic stress $p^{\prime}$, deviatoric strain rate $\dot{\gamma }$ and volume fraction $\Phi$. In this section, the effects of these factors are studied. Based on Eqs. (13)–(15), the rate-dependent behavior of Yurakucho sand for different effective isotropic stresses $p^{\prime }$ are simulated with the parameters calibrated in Table 1. $q^{v} /q_{c}^{s}$ for different effective isotropic stresses $p^{\prime }$ and deviatoric strain rates $\dot{\gamma }$ are then obtained.

**Figure 4:**
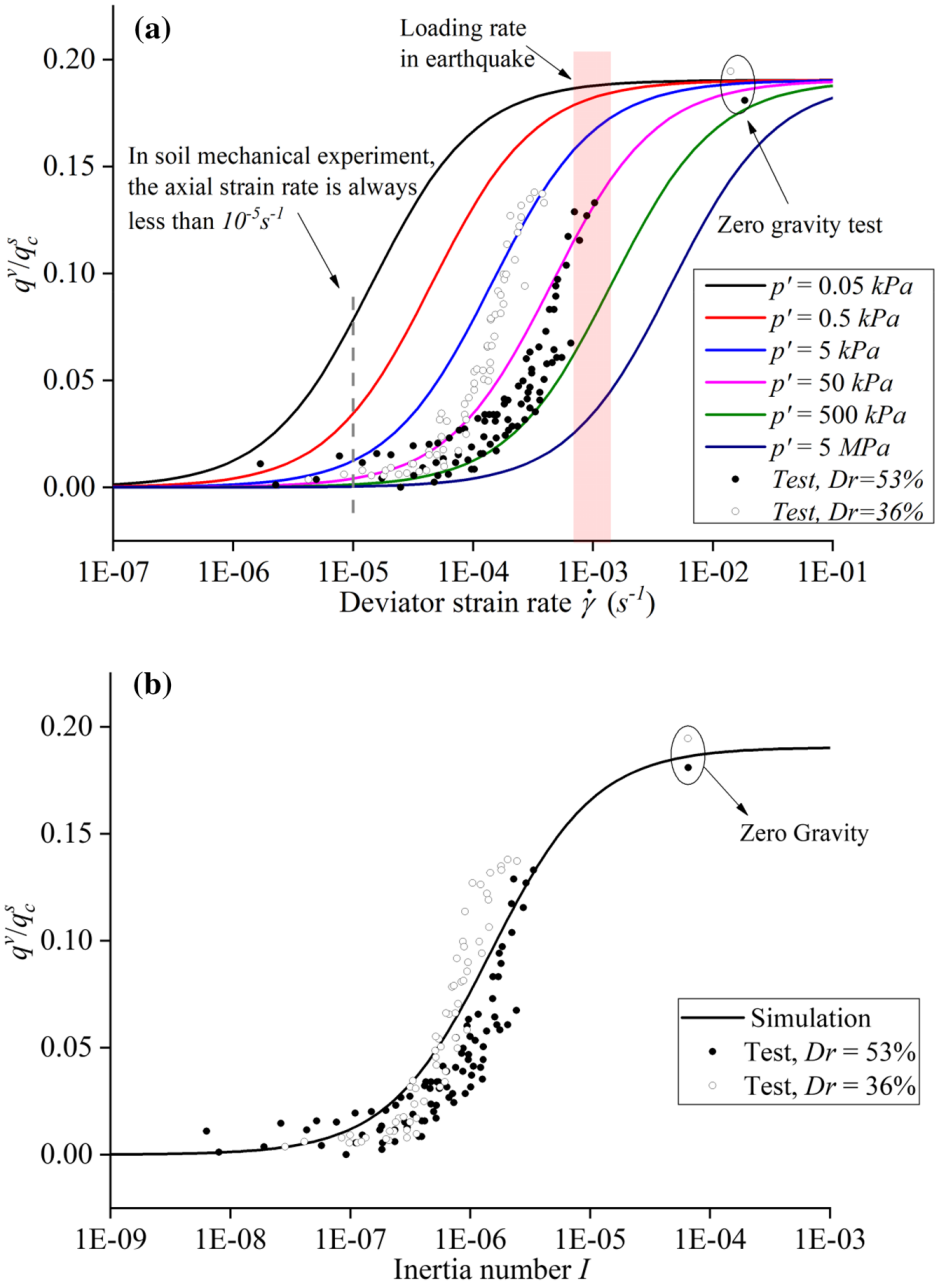
(**a**) Simulated responses of how effective isotropic stresses $p^{\prime }$ and deviatoric strain rates $\dot{\gamma }$ affect ratio of viscous and critical quasi-static stress $q^{v} /q_{c}^{s}$. (**b**) Simulated responses of how effective Inertial number affect ratio of viscous and critical quasi-static stress $q^{v} /q_{c}^{s}$.

In general soil mechanics experiments, the effective isotropic stress $$p^{\prime }$$ is in the range between $$50\;{\text{kPa}}$$ and several thousand $${\text{kPa}}$$, and the deviatoric strain rate $$\dot{\gamma }$$ is less than $$10^{ - 5} {\text{s}}^{ - 1}$$. As shown in Fig. 4a, when the deviatoric strain rate $$\dot{\gamma }$$ is less than $$10^{ - 5} {\text{s}}^{ - 1}$$ of a quasi-static strain rate commonly adopted in soil mechanics lab, $$q_{v} /q_{c}^{s}$$ approaches zero. It implies that the viscous component $$q^{v}$$ is negligible compared to the critical quasi-static component $$q_{c}^{s}$$, and is consistent with the perception that sand is rate-independent in general soil mechanics experiments. The corresponding inertial number in general soil mechanics experiments can be calculated as around 10^–8^ based on the Eq. (4) and Table 1. When the deviatoric strain rate $$\dot{\gamma }$$ is higher than $$10^{ - 4} {\text{s}}^{ - 1}$$ or the effective stress level is decreased in orders, the viscous component $$q^{v}$$ is no longer negligible as the inertial number increases in a few orders. The effects of both effective isotropic stress and strain rate can be alternately characterized by the dimensionless Inertial number in the range between $$10^{ - 8} $$ and $$10^{ - 4} $$ shown in Fig. 4b.

If the effective isotropic stress $p^{\prime}$ is reduced to less than 0.5 kPa, the range in which the viscous component $q^{v}$ become significant is shifted towards smaller deviatoric strain rates $\dot{\gamma }$. The increasing significance of the viscous component $q^{v}$ for low effective isotropic stresses $p^{\prime }$ implies that the sand is no longer rate-independent in such conditions. Earthquake induced liquefaction typically cause deviatoric strain rates of about $\dot{\gamma } \approx 10^{ - 3} {\text{s}}^{ - 1}$. Thus, the viscous component becomes non-negligible and may severely affect underground structures such as tunnels, pipelines, pile foundations and more. Experimental data from triaxial creep tests at low effective isotropic stresses $p^{\prime}$ obtained by^19^ are reproduced in Fig. 4b. The experimental data fit the model predictions in general tendency fairly well.

### Rate-controlled triaxial shear flow tests

In the previous sections, creep properties of sands were observed in stress-controlled experiments and simulations. In this section, rate-dependent properties of sands are studied by the proposed model using experimental data by Enomoto et al.^25^.

**Figure 5:**
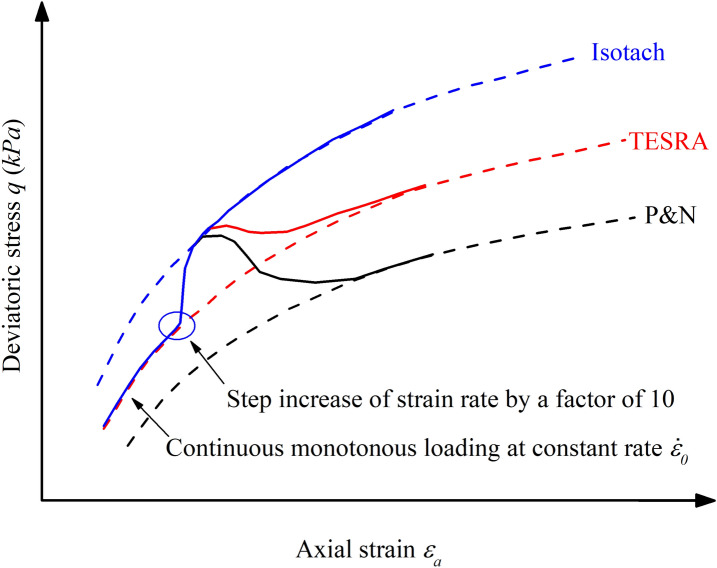
Illustration of common granular viscosity types in sand or other granular materials, namely Isotach, TESRA and P&N.

Sand exhibits different viscous behavior when subject to different deviatoric strain rates $\dot{\gamma }$. In experiments with varying deviatoric strain rates $\dot{\gamma }$, different types of sands will have unique viscous reactions which can be seen in stress–strain curves. The rate-dependent behavior of sand can be classified into viscosity types such as Isotach, TESRA (Temporary Effects of Strain Rate and Acceleration) and P&N (Positive and Negative). The three mentioned types of granular viscosity can be seen in Fig. 5.

The most common granular viscosity type is Isotach viscosity. If Isotach sands are subjected to an instantaneous increase in strain rates, the stress path will also increase instantaneously and follow a new and higher stress path. For TESRA viscosity, the stress path does not exhibit the same uniquely defined stress path as Isotach viscosity. If TESRA sands are subjected to an instantaneous increase in strain rate, the stress path will only temporarily follow a higher stress path before it reverts to the original stress path independent of the new strain rate. For P&N viscosity, the stress path may fall to a lower stress path after an instantaneous increase in strain rate. However, P&N viscosity is rare and has not been explicitly confirmed by experiments.

Enomoto et al. performed multiple unconfined torsional shear and triaxial shear experiments on Miho and Toyoura sand. In this section, relaxation and creep behavior in triaxial shear experiments with the proposed model on Miho and Toyoura sand are simulated. The simulations will be compared with experimental results from Enomoto et al. Material parameters of Miho and Toyoura sand are shown in Table 1.

**Figure 6:**
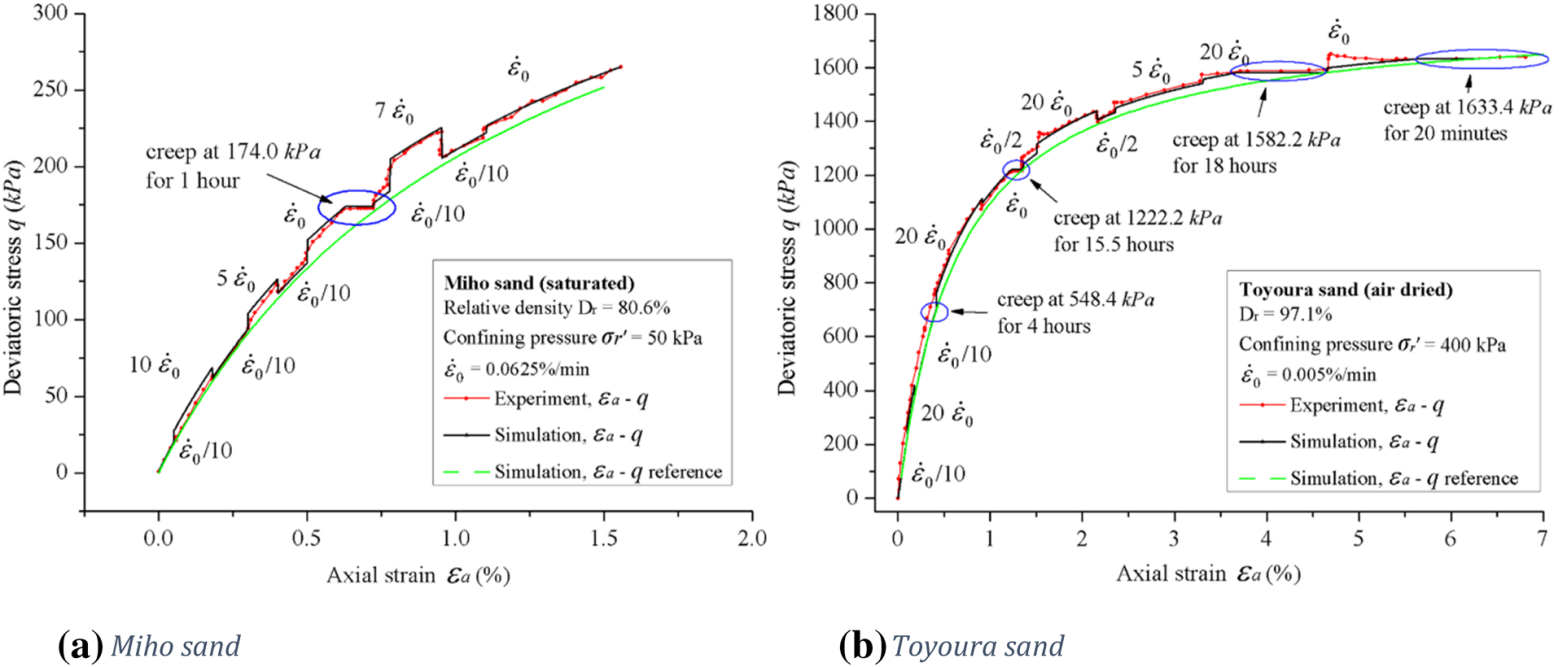
Experimental results of strain-rate controlled triaxial shear experiments by Enomoto et al. (**a** Miho sand; **b** Toyoura sand).

Figure 6 shows both the simulated relationship between the deviatoric stress $q$ and the axial strain $\varepsilon_{a}$, and experimental results from Enomoto et al. for a strain-rate controlled triaxial shear experiment. In both figures the red dotted lines are experimental results, the black solid lines are simulations from the model and the green solid line is the simulated quasi-static stress–strain relationship.

Figure 6a shows the experimental results and simulations for Miho sand with an effective confining pressure of 50 kPa. In the experiment, the axial strain rate ${\dot{\varepsilon }}_{{\text{a}}}$ is varied in the interval $0.1{\dot{\varepsilon }}_{0} \le {\dot{\varepsilon }}_{{\text{a}}} \le 10{\dot{\varepsilon }}_{0}$ where the reference strain rate ${\dot{\varepsilon }}_{0}$ is defined as ${\dot{\varepsilon }}_{0} = 0.0625{\text{ \% min}}^{ - 1} = 1.04 \times 10^{ - 5} \;{\text{s}}^{ - 1}$. When the axial strain rate $\dot{\varepsilon }_{a}$ is $0.1\dot{\varepsilon }_{0}$, the experimental results and the quasi-static reference curve coincides, indicating that the rate-dependent viscous component $q^{v}$ is small. When the axial strain rate $\dot{\varepsilon }_{a}$ changes suddenly with a magnitude ${\Delta }\dot{\varepsilon }_{a}$, the deviatoric stress $q$ changes accordingly with a magnitude ${\Delta }q$. The change in deviatoric stress ${\Delta }q$ shows a strong positive correlation with the change in axial strain rate ${\Delta }\dot{\varepsilon }_{a}$ which is typical Isotach behavior as described in Fig. 5 as any positive change of the axial strain rate $\dot{\varepsilon }_{a}$ will significantly change the viscous component $q^{v}$ defined in Eq. (15).

When the deviatoric stress $q$ reaches 174.0 kPa, the deviatoric stress $q$ is kept constant and the sand is creeped for one hour. The axial strain $\varepsilon_{a}$ gradually approaches the reference curve during the one-hour creep. After the one-hour creep, loading is resumed with an axial strain rate $\dot{\varepsilon }_{a} = \dot{\varepsilon }_{0}$. The stress path makes an immediate jump and follows a new and higher stress path.

It is shown that the model simulates Isotach behavior of Miho sand well. The rate-dependency of Miho sand in the simulation is consistent with experimental results at different strains. The change in deviatoric stress ${\Delta }q$ is strongly correlated with the change of axial strain rate ${\Delta }\dot{\varepsilon }_{a}$, and the magnitude of the change in deviatoric stress ${\Delta }q$ does not decay with continued axial strain $\varepsilon_{a}$.

Figure 6b shows the experimental results and simulations for Toyoura sand with an effective confining pressure of 400 kPa. In the experiment, the axial strain rate $\dot{\varepsilon }_{a}$ is varied in the interval $0.1\dot{\varepsilon }_{0} \le \dot{\varepsilon }_{a} \le 20\dot{\varepsilon }_{0}$ where the reference strain rate $\dot{\varepsilon }_{0}$ is defined as $\dot{\varepsilon }_{0} = 0.005 {\text{\% min}}^{ - 1} = 8.33 \times 10^{ - 7} \;{\text{s}}^{ - 1}$. But from Fig. 6b, the experimental and simulated results show that Toyoura sand exhibit weak Isotach behavior. The discrepancy between the experimental and simulated curves are small, and there is no distinct rate-dependent viscous component $q^{v}$.The absence of a distinct rate-dependent viscous component $q^{v}$ suggests that Toyoura sand with an effective confining pressure of 400 kPa is TESRA viscous. However, the sudden increase in the deviatoric stress ${\Delta }q$ and corresponding increase in axial strain rate ${\Delta }\dot{\varepsilon }_{a}$ is not well simulated. This is to be studied further. The limitation of this model does not affect its overall rate-independency for TESRA viscous materials.

### Pressure dependent viscosity of granular materials

In the two previously presented experiments, Toyoura sand showed different transient flow behavior. In the triaxial creep experiment with low effective isotropic stress by Towhata et al., significant granular viscosity was demonstrated. In Enomoto et al.’s experiment, Toyoura sand exhibited much less viscosity behavior with TESRA viscosity when subject to an effective isotropic stress of 400 kPa. It is generally believed that Isotach and TESRA behavior of sands are determined mainly by intrinsic material properties. However, no clear physical mechanism and quantitative description are explicitly agreed upon. In the model presented in this paper, different viscous behavior of granular materials is highly sensitive to the effective stress level shown in Fig. 4. The model simulations uncover the pressure dependent physical mechanism of viscosity of granular materials.

**Figure 7:**
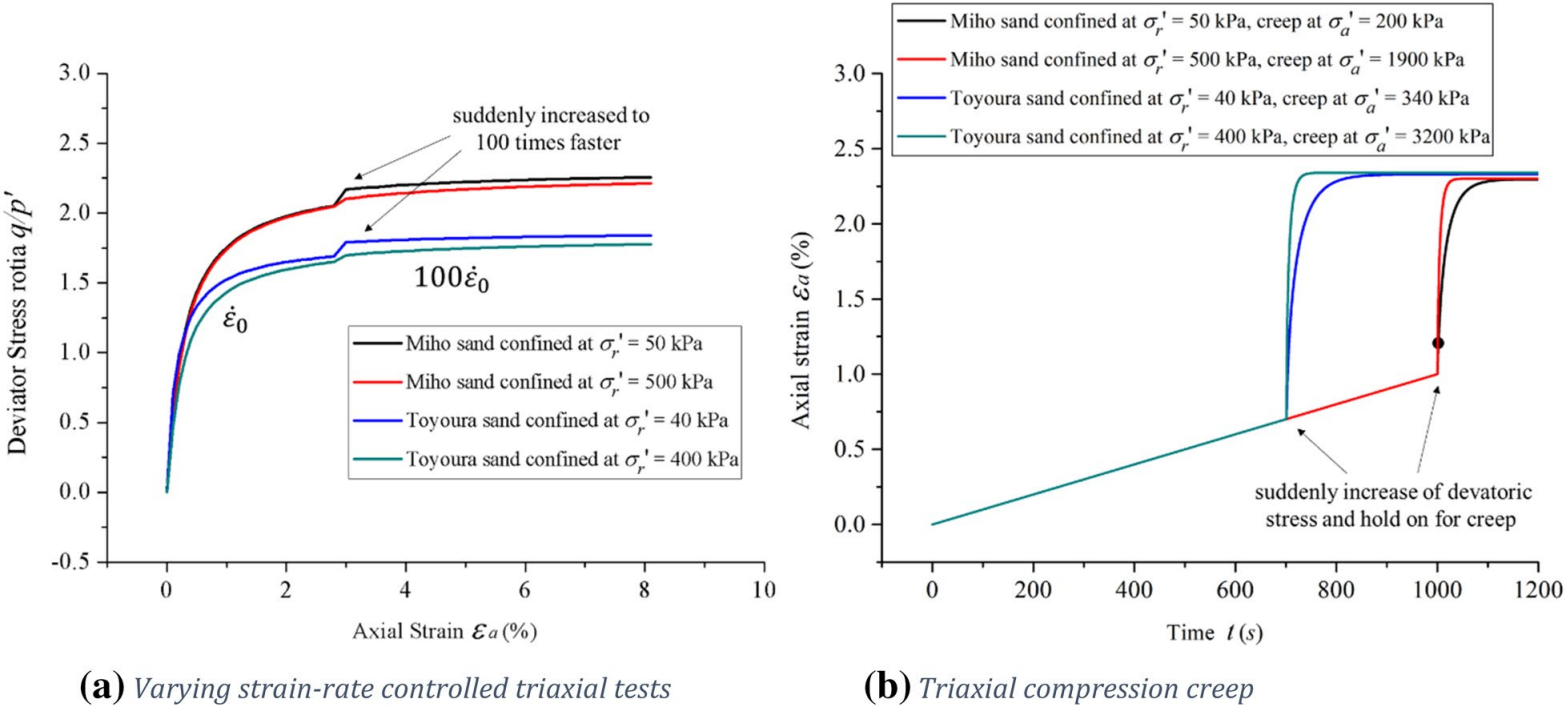
The influence of effective isotropic stresses on the viscosity type of rate-dependent behavior of sands. Red and green lines represent weak Isotach or TESRA behavior, whilst blue and black lines represent strong Isotach behavior.

Based on proposed model in this paper, simulations in Fig. 7 show that when the effective confining pressure of Miho sand is raised from 50 to 500 kPa, the flow behavior will make transition from strong Isotach to weak Isotach or TESRA for both varying triaxial strain rates and creep tests. In Fig. 7a, the strain rate is suddenly increased to 100 times faster, in Fig. 7b, the deviatoric stress is suddenly increased and held on to measure the creeping. Similarly, when the effective stress of Toyoura sand is reduced from 400 to 40 kPa, the rate-dependent behavior will make transition from weak Isotach or TESRA to strong Isotach.

Observe that the flow behavior of sand cannot be singly attributed to material properties, but that it is also the result of granular interaction properties which are dependent on external parameters such as the effective isotropic stress. In the description of this model, the inertial number $I$ reflects the influence of the effective isotropic stress on the viscosity behavior of granular materials. Sands under very low effective isotropic stresses may respond in a pressure sensitive viscous and not quasi-static manner, even if the loading rate is very low. The described response is due to the low natural frequency and increased viscosity in such conditions. This behavior is believed to be responsible for the large peak friction angles observed by Sture et al. in the NASA-MGM program.

## Conclusions

In this paper, the proposed model has demonstrated a good ability to accurately simulate pressure sensitive shear flow behavior of granular materials under the conditions of isotropy and moderate density of the sample. This has been achieved by separating the total stress $\tau_{ij}$ into a viscous part $\tau_{ij}^{v}$ described by the viscous friction coefficient $\mu_{v}$, and a quasi-static component $\tau_{ij}^{s}$ described by the mobilized friction coefficient $\mu_{m}$. Both the viscous friction coefficient $\mu_{v}$ and the mobilized friction coefficient $\mu_{m}$ are quantified by extensive experimental data.

We propose that the pressure sensitivity of the granular viscous component $\tau^{v}$ exists in all transient and steady flow states. Based on the quasi-static component $\tau^{s}$, the viscous component $\tau^{v}$ must always be considered to determine whether the viscous mechanism is of significance or not. In our proposed model, the viscous component $\tau^{v}$ is generally neglected in quasi-static loading when the deviatoric strain rate $\dot{\gamma }$ is equal to or smaller than $10^{ - 4} s^{ - 1}$ for the effective isotropic stresses used in laboratories.

In traditional studies, the flow behavior of granular materials is mainly attributed to material properties. However, lower effective isotropic stresses $p^{\prime}$ may increase the effective friction coefficient significantly which can result in a non-negligible viscous component $\tau^{v}$. The granular viscosity of Isotache behavior in low effective isotropic stresses can occur for granular materials with the presence of a non-negligible viscous stress component $\tau^{v}$. This should be accounted for when designing buried infrastructure against earthquake induced liquefaction. The pressure sensitive granular viscosity found in this paper has implications on the understanding of large peak friction angles found in NASA-MGM experiments for extremely low effective isotropic stresses.

In the field of granular physics, the MiDi rheological model describes a steady dense granular flow. The MiDi rheological model cannot be directly applied to problems concerning transient flows such as creep and strain rate-controlled shear flow and earthquake liquefaction. Simulations and experiments on sands in this paper for low effective isotropic stresses and varying strain rates, have revealed that the sands and other granular materials also exhibit Isotach behavior pre-critical state. The sensitivity of the experiments to low effective isotropic stresses are in general neglected in the experiments. Understanding and modelling transient flows of sand starting from pre-critical states will have a profound influence in engineering.

For problems concerning transient behavior of sands such as earthquakes, landslides, dynamic actions on subsea structures, granular phase-transition problems and problems with high deviatoric strain rates $\dot{\gamma }$ or low effective isotropic stresses $p^{\prime}$, the proposed model is more accurate than current accepted constitutive models, most of which are either rate independent or in the steady state. For the experimental data in Fig. 4, the total stress will be underestimated up to 20% using rate independent model. The original MIDI model is not capable of modeling the Fig. 4 data. Meanwhile the proposed model highlights the role of effective stress in the granular shear flow transitions under the conditions of isotropy and moderate density of the sample. We believe the uncovered new physical mechanism in our proposed model should be valid for sand as well as other granular systems. However, quantitative verification of the proposed model for different scenarios and different granular systems must be carried out to clarify its limitations and relevance to different problems in constitutive or representative element volume and boundary-valued configurations.
